# Replication and pathogenic potential of influenza A virus subtypes H3, H7, and H15 from free-range ducks in Bangladesh in mammals

**DOI:** 10.1038/s41426-018-0072-7

**Published:** 2018-04-25

**Authors:** Rabeh El-Shesheny, Mohammed M. Feeroz, Scott Krauss, Peter Vogel, Pamela McKenzie, Richard J. Webby, Robert G. Webster

**Affiliations:** 1 Department of Infectious Diseases, St. Jude Children’s Research Hospital, Memphis, TN USA; 2 Center of Scientific Excellence for Influenza Viruses, National Research Centre, Giza, Egypt; 3 Department of Zoology, Jahangirnagar University, Dhaka, 1342 Bangladesh; 4 Department of Pathology, St Jude Children’s Research Hospital, Memphis, TN USA

## Abstract

Surveillance of wild aquatic birds and free-range domestic ducks in the Tanguar Haor wetlands in Bangladesh has identified influenza virus subtypes H3N6, H7N1, H7N5, H7N9, and H15N9. Molecular characterization of these viruses indicates their contribution to the genesis of new genotypes of H5N1 influenza viruses from clade 2.3.2.1a that are dominant in poultry markets in Bangladesh as well as to the genesis of the highly pathogenic H5N8 virus currently causing disease outbreaks in domestic poultry in Europe and the Middle East. Therefore, we studied the antigenicity, replication, and pathogenicity of influenza viruses isolated from Tanguar Haor in the DBA/2J mouse model. All viruses replicated in the lung without prior mammalian adaptation, and H7N1 and H7N9 viruses caused 100% and 60% mortality, respectively. H7N5 viruses replicated only in the lungs, whereas H7N1 and H7N9 viruses also replicated in the heart, liver, and brain. Replication and transmission studies in mallard ducks showed that H7N1 and H7N9 viruses replicated in ducks without clinical signs of disease and shed at high titers from the cloaca of infected and contact ducks, which could facilitate virus transmission and spread. Our results indicate that H7 avian influenza viruses from free-range ducks can replicate in mammals, cause severe disease, and be efficiently transmitted to contact ducks. Our study highlights the role of free-range ducks in the spread of influenza viruses to other species in live poultry markets and the potential for these viruses to infect and cause disease in mammals.

## Introduction

Wild aquatic birds are the primary natural reservoir for influenza A viruses (IAVs)^1^. They play a major role in the global distribution of IAVs and the emergence of novel IAVs, which can affect human and animal health worldwide^2, 3^. IAVs are classified on the basis of surface glycoproteins hemagglutinin (HA) and neuraminidase (NA). Currently, IAVs are classified into 18 HA subtypes (H1–H18) and 11 NA subtypes (N1–N11). Only subtypes H1, H2, and H3 have been associated with human pandemics. However, subtypes H5, H6, H7, H9, and H10 can cross the species barrier from birds to mammals, including humans, and cause sporadic infections, but have not yet acquired the ability to transmit between humans^4–7^. The reasons for this remain unclear.

Domestic ducks can perpetuate most avian influenza viruses (AIVs), asymptomatically shed and spread influenza viruses, and play an important role in epidemiology and reassortment of low-pathogenic avian influenza (LPAI) viruses^1, 8^. This was evident in the emergence of an LPAI H7N9 IAV in China in 2013 that spread to humans in live poultry markets. Epidemiological data indicated that this virus originated in domestic ducks and spread to chickens^9–11^. The recent cases of human infections with H10N8 IAVs in China highlight that domestic ducks at sentinel duck farms act as intermediate hosts^12^. Free-range ducks have also been associated with H5N1 outbreaks^13^. Thus, free-range ducks are considered to be the first line of connection between wild and domestic birds and may be key to the emergence and spread of zoonotic AIVs.

Active surveillance of poultry has been conducted in Bangladesh since November 2008, after the first highly pathogenic avian influenza (HPAI) H5N1 outbreak in 2007. Several influenza virus subtypes, including H5 and H9, have been isolated in live poultry markets^14^. In Bangladesh, *H5-HA* genes of the first H5N1 viruses identified in 2007 belonged to clade 2.2.2. Subsequently, 2 subclades of H5N1 viruses, 2.3.2.1a and 2.3.4.2, were detected. Subclade 2.3.2.1a viruses have been dominant in Bangladeshi poultry since 2011^15, 16^. As of 30 September 2017, 8 laboratory-confirmed human cases of H5N1 in Bangladesh have been reported to the World Health Organization (WHO), of which 1 case was fatal. Circulation of HPAI H5N1 viruses may increase the chances of emergence of pandemic strains through reassortment. H5N1 reassortant viruses containing the polymerase basic 1 (*PB1*) gene from H9N2 viruses have been reported^17, 18^. Recently, we described the emergence of a novel genotype of H5N1 virus that contained the *HA*, matrix (*M*), and *NA* genes of circulating Bangladeshi H5N1 viruses and five genes from Eurasian-lineage LPAI viruses^19^.

Bangladesh is located in the central Asian flyway and is near the Eastern Asian–Australian and Black Sea–Mediterranean flyways. Tanguar haor, a vast wetland in northeastern Bangladesh, also serves as the major wintering ground for birds migrating in both the Central Asian and Eastern Asian–Australian flyways. As this location facilitates contact between wild birds and free-range ducks, surveillance for influenza viruses was conducted in wild birds and free-range ducks in Tanguar haor in Bangladesh in February 2015 and February 2016. Various LPAIV subtypes, including H3, H7, and H15, have been isolated in the Tanguar haor area. Our previous study showed genetic similarities between internal gene segments of these viruses with some genes of HPAI H5N8 clade 2.3.4.4 origin, suggesting their potential role in the genesis of the HPAI A(H5N8) virus that emerged in 2016^20^. Therefore, we sequenced the full genomes of H3, H7, and H15 AIVs and analyzed their genetic and antigenic characteristics to better understand the evolution of these influenza viruses that have played important roles in the emergence of the new H5N1 genotype currently circulating in Bangladesh and in the genesis of H5N8 circulating in Europe and the Middle East. We also evaluated the replication and pathogenicity of these isolates in mice and mallard ducks.

## Results

### Isolation and IVPI of influenza viruses from wild birds and free-range ducks

Thirteen AIVs were isolated from wild birds and free-range flocks of domestic ducks in the Tanguar haor region in February 2015 and February 2016. Four influenza A (H3N6), four influenza A (H7N1), one influenza A (H7N5), three influenza A (H7N9), and two influenza A (H15N9) viruses were isolated from free-range flocks of domestic ducks. Also, an influenza A (H7N5) virus was isolated from a migratory black-tailed godwit. The intravenous pathogenicity index (IVPI) of the seven viruses (A/duck/Bangladesh/26920/2015 H3N6, A/duck/Bangladesh/26948/2015 H3N6, A/duck/Bangladesh/26918/2015 H3N6, A/duck/Bangladesh/24692/2015 H7N1, A/black-tailed godwit/Bangladesh/24734/2015 H7N5, A/duck/Bangladesh/26980/2015 H7N9, and A/duck/Bangladesh/24697/2015 H15N9) ranged from 0.0 to 0.05 in chickens (Supplementary Table S1),

### Antigenic characterization

To determine antigenic relationships among influenza viruses isolated from free-range ducks and a wild bird, we analyzed H3, H7, and H15 viruses by using a panel of polyclonal and monoclonal antibodies (MAbs) against Eurasian and North American viruses. Antigenic analysis showed that H3 viruses were homogenous and similar to the Eurasian lineage. Three H3 viruses reacted with the antiserum against A/duck/Shantou/1283/2001 (H3N8) at higher titers than for other antisera against viruses from the Eurasian lineage (Table 1). Antigenic relationships between H7 viruses were determined by using ferret antisera. Antisera against these viruses cross-reacted well together, demonstrating the antigenic similarity of H7 viruses (Table 2). A panel of MAbs recognizing antigenic sites I and II on the HA protein of A/seal/Massachusetts/1/1980 (H7N7) (Supplementary Table S2) and two MAbs recognizing an antigenic site on A/chicken/Victoria/1985 (H7N7) were used. All MAbs reacted well with all H7N1, H7N5, and H7N9 isolates, indicating that antigenic sites I and II of A/seal/Massachusetts/1/1980 (H7N7) were conserved in all H7 viruses (Table S2). Antigenic analysis with polyclonal antisera revealed that two isolates (H15N9) were antigenically distinct from the reference A/duck/Australia/341/1983(H15N8) strain. Further, antiserum raised against A/duck/Australia/341/1983 (H15N8) did not react well with Bangladesh viruses (H15N9) (Supplementary Table S3).

**Table 1:** Antigenic characterization by the hemagglutination inhibition assay of H3 viruses isolated from free-range ducks in Bangladesh

	Antisera						
	North American	Eurasian				Australian	Bangladeshi
*Reference virus*	*IN/0392 H3N2*	*Hong Kong/1/68*	*Ukraine/1/63*	*Shantou/1283/2001*	*Bulgaria/61/2008*	*South Australia/55/2014*	*A/duck/BD/26920/2015 H3N6*
A/swine/Indiana/0392/2011 H3N2	**2560**	<10	<10	40	<10	<10	<10
A/Hong Kong/1/68 H3N2	20	**10,240**	2560	10,240	640	<10	2560
A/duck/Ukraine/1/63 H3N8	<10	160	**40**	1280	80	<10	160
A/duck/Shantou/1283/2001 H3N8	<10	160	80	**640**	40	<10	160
A/muleduck/Bulgaria/61/2008 H3N2	<10	80	40	320	**80**	<10	320
A/South Australia/55/2014 H3N2	<10	20	<10	<10	<10	**160**	<10
A/Aichi/2/68 H3N2	<10	2560	1280	2560	160	<10	2560
*Test virus*							
A/duck/Bangladesh/26920/2015 H3N6	<10	80	80	640	80	<10	**640**
A/duck/Bangladesh/26948/2015 H3N6	<10	80	40	320	40	<10	320
A/duck/Bangladesh/26918/2015 H3N6	<10	80	80	640	80	<10	640

Homologous titers are bold underlined

**Table 2:** Antigenic characterization by the hemagglutination inhibition assay of H7 viruses isolated from free-range ducks in Bangladesh

	Antisera							
	North American		Eurasian					
*Reference viruses*	*A/Canada/RV444/2004*	*A/chicken/Saskatchewan/HR10/2007 H7N3*	*A/equine/Prague/56 H7a*	*A/FPV/Rostock H7b* ^a^	*A/Netherlands/219/2003 H7N7*	*Anhui/1/2013 H7N9*	*A/duck/Bangladesh/24692/2015 H7N1*	*A/duck/Bangladesh/26980/2015 H7N9*
RG-A/Canada/RV444/2004 H7N3	**80**	40	<10	5120	40	20	640	1280
A/equine/Prague/56 H7a	40	10	**320**	5120	40	10	160	160
RGA/Netherlands/219/2003 H7N7	40	20	<10	2560	**20**	10	320	640
RG-A/Anhui/1/2013 H7N9	80	40	<10	5120	320	**80**	1280	1280
A/seal/MA/1/80 H7N7	80	40	<10	5120	20	20	640	640
A/Ruddy turnstone/NJ/65/85 H7N3	40	10	<10	1280	10	10	320	320
*Test virus*								
A/duck/Bangladesh/24692/2015 H7N1	40	20	<10	2560	10	10	**320**	640
A/duck/Bangladesh/24694/2015 H7N1	40	40	<10	2560	20	20	320	160
A/duck/Bangladesh/24705/2015 H7N1	80	20	<10	5120	20	10	320	80
A/duck/Bangladesh/24706/2015 H7N1	40	20	<10	2560	10	20	320	80
A/black-tailed godwit/Bangladesh/24734/2015	40	20	<10	2560	10	10	320	160
*H7N5*								
A/duck/Bangladesh/26980/2015 H7N9	80	40	<10	2560	20	10	320	**320**
A/duck/Bangladesh/26992/2015 H7N9	40	20	<10	2560	10	10	320	80
A/duck/Bangladesh/27042/2015 H7N9	40	20	<10	2560	20	10	320	160

Homologous titers are bold underlined

^a^ Goat hyperimmune sera

### Pathogenicity in DBA/2J mice

**Fig. 1:**
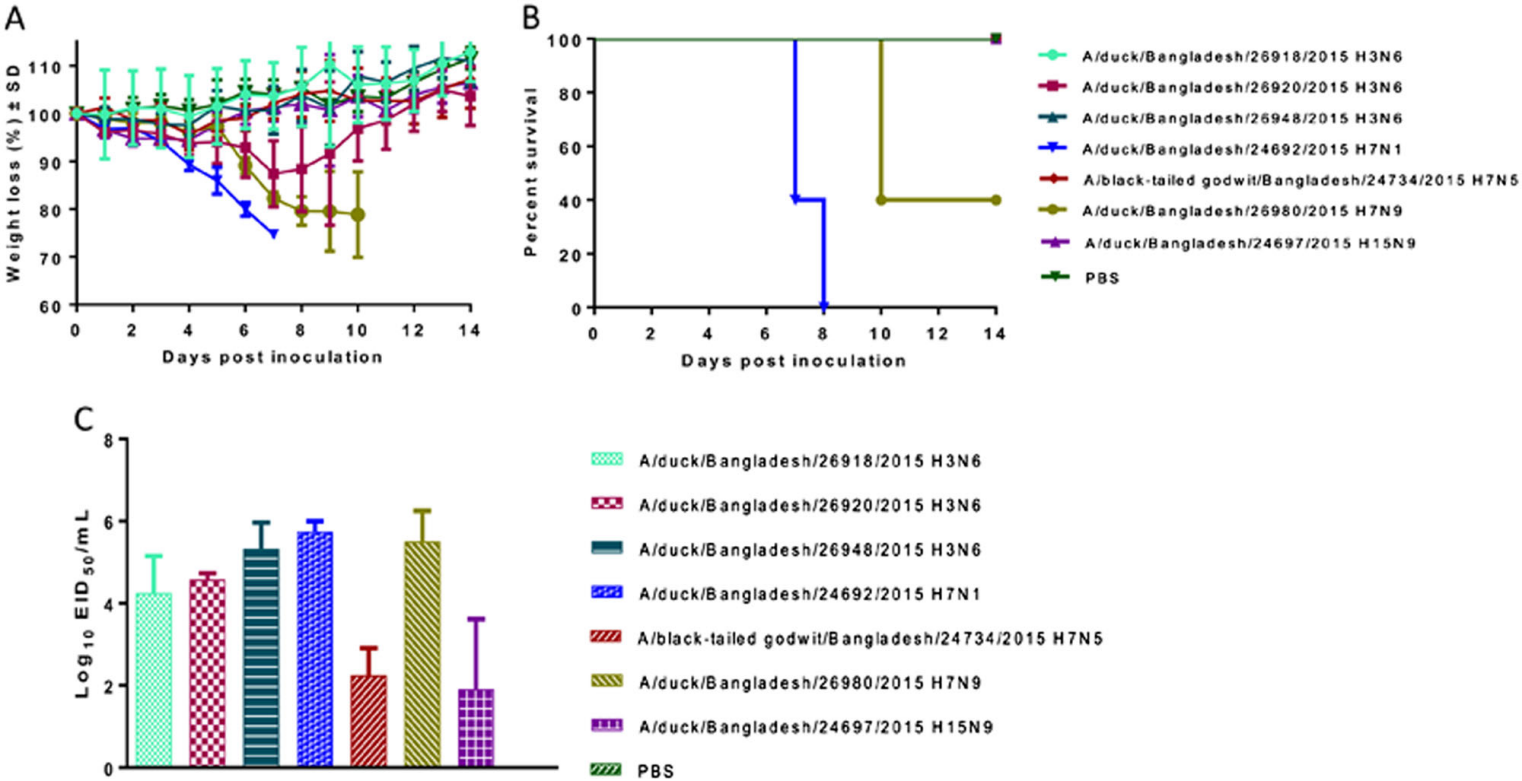
Percent weight loss and survival and lung titration in DBA/2J mice infected with 10^6^ EID_50_ of selected isolates from free-range ducks and wild birds. **a** Daily weight loss was calculated as the percentage of weight relative to that at 0 dpi, and mean weight loss for each group is shown. **b** Mortality was recorded based on actual death or killing at 25% weight loss, according to our protocol. For each virus isolate, five mice were infected. **c** Lung tissues were collected at 3 dpi, and viral titers were determined by EID_50_ assays. Error bars indicate standard deviation, and the dotted line indicates the lower limit of detection of the infectious virus

To determine the ability of influenza subtypes isolated from free-range ducks and a wild bird to replicate and cause disease in mammals, the pathogenicity of 3 H3N6, 1 H7N1, 1 H7N5, 1 H7N9, and 1 H15N9 viruses was studied in DBA/2J mice. The DBA/2J mice were inoculated with 10^6^ egg infectious dose (EID_50_) of each virus in 30 μL of phosphate-buffered saline (PBS) (*n* = 5 mice per virus isolate), and morbidity and mortality were monitored daily for 14 day post inoculation (dpi) (Fig. 1). A/duck/Bangladesh/24692/2015 (H7N1) and A/duck/Bangladesh/26980/2015 (H7N9) viruses caused 100% and 60% mortality, respectively, whereas the other five viruses did not cause mortality. On the basis of these findings, A/duck/Bangladesh/24692/2015 (H7N1) was classified as pathogenicity index (PI) PI-4, A/duck/Bangladesh/26980/2015 (H7N9) was classified PI-2, and the remaining viruses were classified PI-0 (Table 3). To determine virus replication in mice, lung tissue samples were collected from three killed mice per group at 3 dpi. All seven viruses replicated in the lungs of DBA/2J mice. A/duck/Bangladesh/24692/2015 (H7N1) and A/duck/Bangladesh/26980/2015 (H7N9) viruses induced high morbidity and mortality that correlated with high viral titers in the lungs (4.75 log_10_–6.25 log_10_ EID_50_/mL) (Fig. 1). These results indicate that all seven viruses replicate well in mammals without prior adaptation.

**Table 3:** Pathogenicity in DBA/2J mice infected with avian viruses isolated from free-range ducks and wild birds

Influenza strain	Subtype	Survival (AUC)	Weight loss (AUC)	Survival score^a^	Weight loss score^b^	Final score^c^	PI
A/duck/Bangladesh/26918/2015	H3N6	1400	1469.07	0.800	0.210	1.010	0
A/duck/Bangladesh/26920/2015	H3N6	1400	1342.33	0.800	0.192	0.992	1
A/duck/Bangladesh/26948/2015	H3N6	1400	1447.08	0.800	0.207	1.007	0
A/duck/Bangladesh/24692/2015	H7N1	640	646.24	0.366	0.092	0.458	4
A/black-tailed godwit/Bangladesh/24734/2015	H7N5	1400	1422.82	0.800	0.203	1.003	0
A/duck/Bangladesh/26980/2015	H7N9	1100	1051.84	0.629	0.150	0.779	2
A/duck/Bangladesh/24697/2015	H15N9	1400	1402.67	0.800	0.200	1.000	0
PBS		1400	1454.62	0.800	0.208	1.008	0

*AUC* area under the curve, *PBS* phosphate-buffered saline, *PI* pathogenicity index

^a^ Survival score was calculated as 0.86 (survival AUC/maximum AUC)

^b^ Weight loss score was calculated as 0.26 (weight loss AUC/maximum AUC)

^c^ Total pathogenicity score was the sum of survival and weight loss scores

### Replication kinetics in lung and extrapulmonary tissues in DBA/2J mice

To assess replication kinetics in lungs and other organs, mice were inoculated with one of the three viruses with different PIs—A/duck/Bangladesh/24692/2015 (H7N1) (PI-4), A/duck/Bangladesh/26980/2015 (H7N9) (PI-2), and A/black-tailed godwit/Bangladesh/24734/2015 (H7N5) (PI-0)—and viral replication in lungs, liver, heart, brain, and intestines was determined at 2, 4, 6, and 8 dpi, respectively. All three viruses replicated in the lungs. A/duck/Bangladesh/24692/2015 (H7N1) (PI-4) and A/duck/Bangladesh/26980/2015 (H7N9) (PI-2) had higher shedding than did A/black-tailed godwit/Bangladesh/24734/2015 (H7N5) (PI-0), with average viral titers in the lungs for each group ranging from 10^1.83^/mL to 10^6.42^/mL EID_50_ (Table 4). The lowest lung titer (10^1.83^/mL EID_50_) was detected at 8 dpi in mice infected with A/black-tailed godwit/Bangladesh/24734/2015 (H7N5) (PI-0). There was no replication detected in the intestines of mice infected with any of the viruses. A/duck/Bangladesh/24692/2015 H7N1 (PI-4), and A/duck/Bangladesh/26980/2015 (H7N9) (PI-2) replicated in the heart, liver, and brain. Viral titers were detected in the heart, liver, and brain of mice infected with A/duck/Bangladesh/24692/2015 (H7N1) (PI-4) at all time points (Table 4). Viral titers were detected in the heart at 2, 6, and 8 dpi (10^0.75^/mL–10^4.5^/mL EID_50_); in the liver at 2 and 6 dpi (10^1.75^/mL–10^3.5^/mL EID_50_); and in the brain at 2, 4, 6, and 8 dpi (10^0.75^/mL–10^2.75^/mL EID_50_) of mice infected with A/duck/Bangladesh/26980/2015 (H7N9) (PI-2) (Table 4). Two viruses that caused mortality were detected in the brain; this result is similar with the previous study on H7 viruses North America^21^. Overall, our results show that viruses isolated from free-range ducks can replicate and some can cause disease in a mammalian model with systemic dissemination.

**Table 4:** Replication kinetics of H7 AIVs in lung and extrapulmonary tissues in mice

Virus	PI	Subtype		Viral titers (log_10_ EID_50_/mL)^a^			
				Day			
				2	4	6	8
A/duck/Bangladesh/24692/2015	4	H7N1	Lung	3/3 (5.67 ± 0.52)	3/3 (5.42 ± 0.88)	3/3 (6.42 ± 0.14)	3/3 (6.17 ± 0.38)
			Heart	2/3 (2.13 ± 0.88)	2/3 (3.00 ± 0.71)	3/3 (2.92 ± 1.13)	2/3 (2.63 ± 0.18)
			Liver	3/3 (2.67 ± 0.52)	1/3 (4.25)	1/3 (2.50)	2/3 (2.50 ± 1.41)
			Intestine	—	—	—	—
			Brain	2/3 (2.13 ± 0.18)	2/3 (1.88 ± 0.53)	1/3 (3.75)	3/3 (2.67 ± 0.52)
A/duck/Bangladesh/26980/2015	2	H7N9	Lung	3/3 (5.50 ± 1.00)	3/3 (5.88 ± 0.53)	3/3 (5.92 ± 0.38)	3/3 (5.25 ± 0.75)
			Heart	2/3 (3.38 ± 1.59)	—	1/3 (2.00)	1/3 (1.75)
			Liver	2/3 (2.50 ± 0.35)	—	2/3 (2.63 ± 1.24)	—
			Intestine	—	—	—	—
			Brain	2/3 (2.25 ± 0.71)	—	1/3 (1.50)	2/3 (2.13 ± 0.18)
A/black-tailed godwit/Bangladesh/24734/2015	0	H7N5	Lung	2/3 (2.88 ± 2.2)	3/3 (2.67 ± 1.01)	3/3 (3.42 ± 1.01)	3/3 (1.83 ± 0.58)
			Heart	—	—	—	—
			Liver	—	—	—	—
			Intestine	—	—	—	—
			Brain	—	—	—	—

^a^ The lower limit of detection of infectious virus was <0.75 log_10_ EID_50_

### Histologic changes in mouse lung tissues

**Fig. 2:**
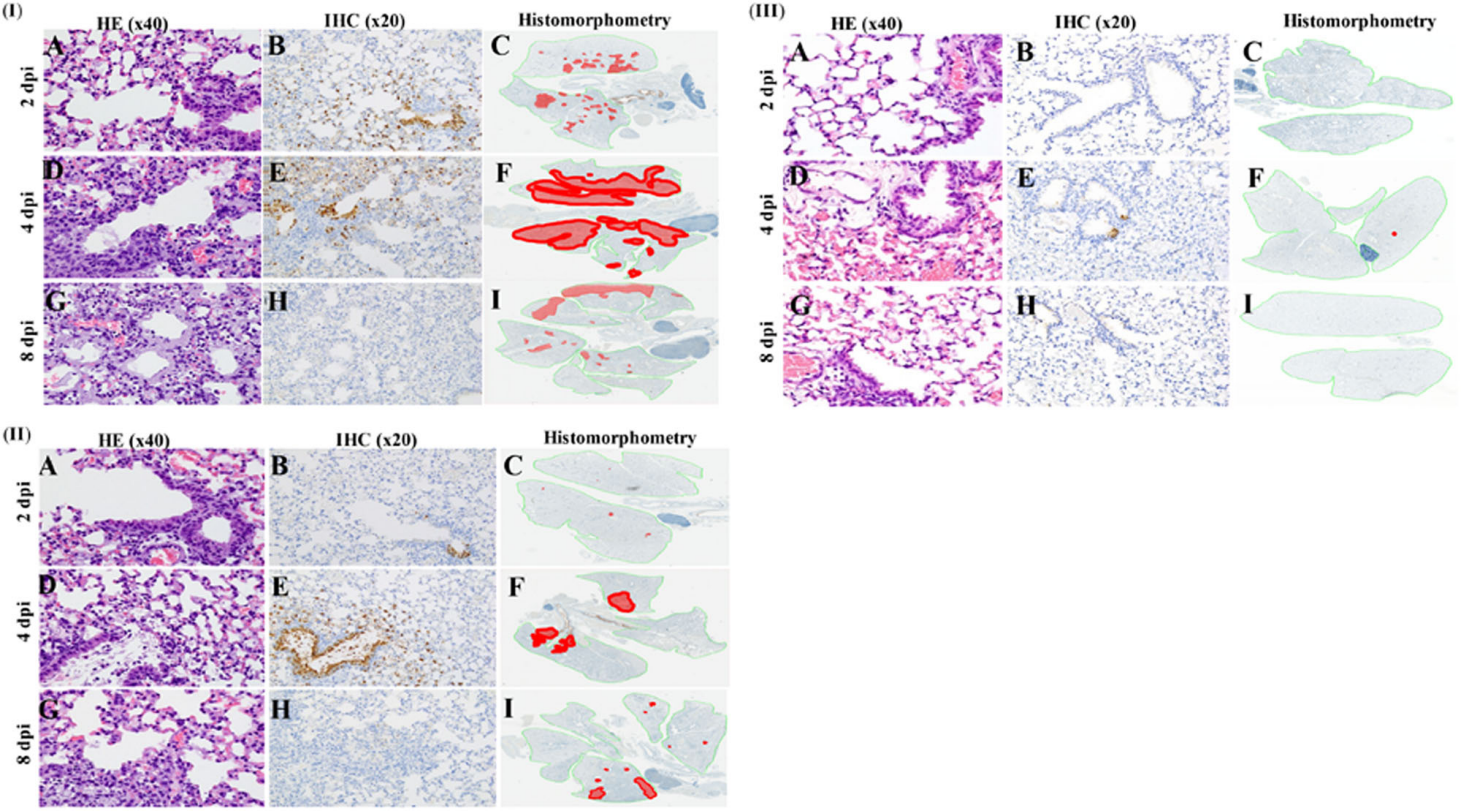
Pulmonary lesions and virus spread in the lung of mice infected with A/duck/Bangladesh/24692/2015 H7N1 (**I** A–I), A/duck/Bangladesh/26980/2015 H7N9 (**II** A–I), and A/black-tailed godwit/Bangladesh/24734/2015 H7N5 (**III** A–I) at 2, 4, and 8 dpi . Mouse lungs were fixed in 10% neutral buffered formalin and stained with hematoxylin-eosin (HE), subjected to immunohistochemical (IHC) staining with anti–NP antiserum, or analyzed by histomorphometry (magnification: ×40 HE, ×20 IHC, and ×2 histomorphometry). In the histomorphometry images, the total lung areas examined are outlined in green; areas of active infection with antigen-positive cells are shown in red

The extent and severity of histopathologic lesions in the lungs of mice infected with H7N1, H7N9, and H7N5 virus isolates correlated with the viruses’ differing levels of pathogenicity. The more rapid and extensive spread of infection in H7N1-infected lungs was evident at 2 dpi. At this early time point, there was no notable pulmonary inflammation (Fig. 2I A) but virus-infected cells were present in both distal airways and surrounding alveoli (Fig. 2I B). Virus-infected areas included most central and many peripheral bronchioles, with infection spreading to surrounding alveoli (Fig. 2I C). By 4 dpi, interstitial inflammation and alveolar proteinosis (Fig. 2I D) associated with antigen-positive cells and debris (Fig. 2I E) extended to peripheral margins of the lung (Fig. 2I F). By 8 dpi, pulmonary lesions were characterized by extensive areas of pulmonary consolidation, hyaline membrane formation (indicative of severe damage to the alveolar-capillary membrane), and intra-alveolar neutrophil infiltrates (Fig. 2I G). Virus-infected cells were not detected in central lesions (Fig. 2I I) but were present in peripheral lesions (Fig. 2I H).

At all time points, the severity and extent of virus infection was less in H7N9-infected lungs than in H7N1-infected lungs. Although H7N9 caused extensive infection of the tracheal epithelium by 2 dpi, there was no notable inflammation in the lung parenchyma (Fig. 2II A) and only minimal spread of infection (Fig. 2II B) from a few infected bronchioles to surrounding alveoli (Fig. 2II C). By 4 dpi, multifocal mild interstitial and alveolar inflammation (Fig. 2II D) was associated with moderate spread of infection from central bronchioles to adjacent alveoli (Fig. 2II E). At 4 dpi, there was still no involvement of peripheral alveoli (Fig. 2II F). At 8 dpi, there were multifocal areas with minimal to mild interstitial inflammation and some plugged bronchioles but no hyaline membranes (Fig. 2II G), indicating less severe damage to alveolar-capillary structures. Viral antigen and infected cells had been cleared from central lesions (Fig. 2II H), and only some small areas of active infection remained in peripheral areas of the lung (Fig. 2II I). In marked contrast, pulmonary lesions were essentially absent at 2, 4, and 8 dpi in H7N5-infected mice (Fig. 2III A, III D, III G). Viral antigen was not detected in any part of the lungs at 2 and 8 dpi (Fig., 2III B, III C, IIIH, III I) and was present in only a single bronchiole and few alveoli (Fig. 2III E, III F).

### Replication and transmission in mallard ducks

To assess the pathogenicity of viruses with high PIs in mice, mallard ducks were inoculated with A/duck/Bangladesh/24692/2015 (H7N1) (PI 4) and A/duck/Bangladesh/26980/2015 (H7N9) (PI 2) at a dose of 10^6^ EID_50_/mL. None of the ducks infected with H7N1 or H7N9 viruses by inoculation or after contact with infected birds died or exhibited clinical symptoms (Table S4). However, all inoculated and contact ducks showed seroconversion against H7N1 or H7N9, as detected by the HI assay at 14 dpi (Table S4). Ducks inoculated with the A/duck/Bangladesh/24692/2015 (H7N1) virus had significantly lower body weights at 3 and 5 dpi (Supplementary Figure S1) than did contact ducks exposed to the same virus.

**Fig. 3:**
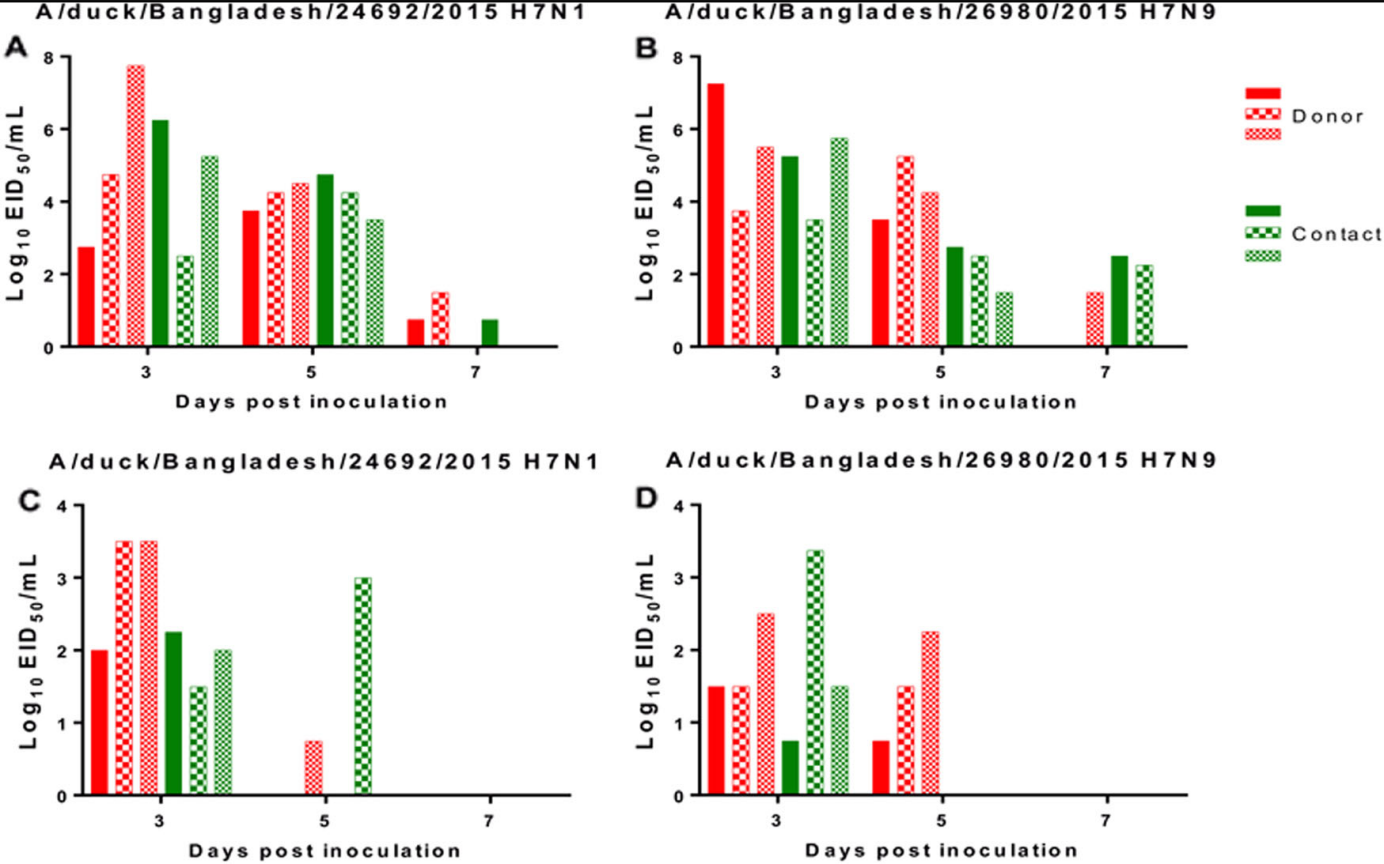
Replication and transmission of H7 viruses in mallard ducks. Viral titers in cloacal (**a**, **b**) and oropharyngeal (**c**, **d**) swabs of mallard ducks infected with A/duck/Bangladesh/24692/2015 H7N1 and A/duck/Bangladesh/26980/2015 H7N9 viruses. Virus shedding of both donor and contact ducks was monitored, and swabs were collected at 3, 5, and 7 dpi and dpc. The dotted line indicates the lower limit of detection of the infectious virus. dpi days post inoculation, dpc days post contact

Replication and transmissibility of these viruses in ducks were evaluated. Cloacal shedding was higher than oropharyngeal shedding in all ducks infected with H7N1 or H7N9 viruses by inoculation or after contact with infected birds. The H7N1 virus was detected in cloacal swabs from inoculated ducks at 3, 5, and 7 dpi (10^0.75^/mL–10^7.75^/mL EID_50_) and from oropharyngeal swabs at 3 and 5 dpi (10^0.75^/mL–10^3.5^/mL EID_50_). The H7N9 virus was detected in cloacal swabs at 3, 5, and 7 dpi (10^1.5^/mL–10^7.25^/mL EID_50_) and from oropharyngeal swabs at 3 and 5 dpi (10^0.75^/mL–10^2.5^/mL EID_50_) (Fig. 3). To determine whether H7N1 and H7N9 viruses were efficiently transmitted between mallard ducks, viruses were isolated from oropharyngeal and cloacal swabs from contact birds (Fig. 3). The H7N1 virus was detected in cloacal swabs from contact ducks at 3, 5, and 7 dpi (10^0.75^/mL–10^6.25^/mL EID_50_) and oropharyngeal swabs at 3 and 5 dpi (10^1.5^/mL–10^3^/mL EID_50_). Also, contact ducks infected with the H7N9 virus had extended shedding from cloacal swabs until 7 dpi (10^1.25^/mL–10^5.75^/mL EID_50_), whereas virus was detected only at 3 dpi in oropharyngeal swabs (10^0.75^/mL–10^3.37^/mL EID_50_).

Overall, viral titers were higher in cloacal swabs than oropharyngeal swabs in ducks infected with H7N1 and H7N9 viruses, but no virus was detected from either infected group after 7 dpi. Virus shedding of H7N1 and H7N9 was detected until 7 dpi in cloacal samples from contact ducks. Virus shedding from oropharyngeal swabs was observed until 5 dpi for both H7N1 and H7N9.

### Amino acid analysis of viruses isolated from free-range ducks and wild birds

To identify amino acids or mutations that may be associated with virulence, pathogenicity, transmission, receptor-binding preference, and drug resistance, we compared the deduced amino acid sequences of the Tanguar haor viruses. The HA segments of four H3 isolates were grouped into the Eurasian lineage and clustered in one genetic group that included H3 viruses from Netherlands, Kazakhstan, Mongolia, and Vietnam^19^. H3N6 isolates shared the same amino acid sequence PEKQTR↓GLF (↓denotes site of protease activity) at the cleavage site between HA1 and HA2, which have been associated with low-pathogenic effects in poultry. All H7 viruses isolated in our study belonged to the Eurasian-lineage H7 viruses, which have two motifs with one arginine residue at the HA cleavage site PETPKGR↓GLF and PELPKGR↓GLF (Table 5). For H15N9 viruses, the cleavage site PEKTHTR↓GLF was similar to that found in the H15N7 virus from Ukraine and H15N4 virus from Russia.

**Table 5:** Comparison of amino acid sequences and IVPI of viruses isolated from free-range ducks in Bangladesh

Strain	Subtype	IVPI	HA			NA			PB2			PB1-F2	NS1
			HA cleavage site	Q226L	G228S	E119V	H275Y	R292K	E627K	D701N	G309D	N66S	C-terminal motif
A/duck/Bangladesh/26920/2015	H3N6	0.0	PEKQTR↓GLF	Q	G	E	H	R	E	D	G	S	ESEV
A/duck/Bangladesh/26948/2015	H3N6	0.05	PEKQTR↓GLF	Q	G	E	H	R	E	D	G	S	ESEV
A/duck/Bangladesh/26974/2015	H3N6	ND	PEKQTR↓GLF	Q	G	E	H	R	E	D	G	S	ESEV
A/duck/Bangladesh/26918/2015	H3N6	0.0	PEKQTR↓GLF	Q	G	E	H	R	E	D	G	N	ESEV
A/duck/Bangladesh/24694/2015	H7N1	0.31	PETPKGR↓GLF	Q	G	E	H	R	E	D	G	N	ESEV
A/duck/Bangladesh/24692/2015	H7N1	0.0	PETPKGR↓GLF	Q	G	E	H	R	E	D	G	S	ESEV
A/duck/Bangladesh/24706/2015	H7N1	ND	PETPKGR↓GLF	Q	G	E	H	R	E	D	G	S	ESEV
A/duck/Bangladesh/24705/2015	H7N1	1.04	PELPKGR↓GLF	Q	G	E	H	R	E	D	G	S	ESEV
A/black-tailed godwit/Bangladesh/24734/2015	H7N5	0.0	PELPKGR↓GLF	Q	G	E	H	R	E	D	G	N	ESEV
A/duck/Bangladesh/26980/2015	H7N9	0.0	PELPKGR↓GLF	Q	G	E	H	R	E	D	G	S	ESEV
A/duck/Bangladesh/26992/2015	H7N9	0.35	PELPKGR↓GLF	Q	G	E	H	R	E	D	G	S	ESEV
A/duck/Bangladesh/27042/2015	H7N9	0.0	PELPKGR↓GLF	Q	G	E	H	R	E	D	G	S	ESEV
A/duck/Bangladesh/24704/2015	H15N9	ND	PEKTHTR↓GLF	Q	G	E	H	R	E	D	D	N	ESEV
A/duck/Bangladesh/24697/2015	H15N9	0.0	PEKTHTR↓GLF	Q	G	E	H	R	E	D	G	N	ESEV

*IVPI* intravenous pathogenicity index, *ND* not done

The HA protein was examined for the presence of N-linked glycosylation motifs, using the N-(P-[S/T]-P) motif to indicate a potential N-linked glycosylation site. The H3N2 viruses had six potential glycosylation sites at positions 22, 38, 165, 285, and 483, whereas H7N1 and H7N5 viruses had five potential glycosylation sites at positions 22, 38, 240, 411, and 483. There were seven potential glycosylation sites for H15N9 viruses at positions 22, 38, 92, 165, 411, 483, and 487. There was a specific insertion in the HA1 subunit at positions 253–262 (SNALSGVEYN) in two isolate H15N9 viruses that characterized the *HA* gene of H15 subtype, which is located in the 260-loop. Q226L and G228S mutations were not detected in the HA protein, and there were no substitutions at residues E190D and G225D, indicating that all virus isolates likely had binding preferences for avian-like α-2,3-linked sialic acid receptors. According to NA sequences, none of the isolates had deletions at amino acid positions 69–73 in the NA stalk region. These viruses also did not display oseltamivir resistance markers E119, H275, R293, and N295 (N1 numbering).

None of the isolated viruses had E627K and D701N mutations in the PB2 protein, which play an important role in the adaptation of AIVs to mammals. A substitution of lysine to glutamine at residue 355 correlates with pathogenicity in mammals^22^, and in our study all isolates had an arginine residue at this location. The PB2 amino acid substitutions E249G, G309D, and T339M increase replication in human lung epithelial cells^23^, all isolates in this study had substitution 309D, except A/duck/Bangladesh/24704/2015 (H15N9) virus. All our isolates expressed PB1-F2 of 90 amino acids. Further, two H7N1 isolates, three H3N6 isolates, and three H7N9 isolates had an N66S mutation in PB1-F2 (Table 5), which increases virulence, replication efficiency, and anti-viral response in mice^24^. A screen for molecular markers of resistance to adamantine at residues L26, V27, A30, S31, and G34 within the M2 protein revealed that all isolates were sensitive to M2 ion channel blockers. Also, there were amino acid substitutions P42S and V149A in the NS1 protein of all isolates. These mutations are associated with virulence and pathogenicity in mammals^25, 26^. For all isolates, the C-terminal motif at residues 227–230 was ESEV.

## Discussion

Wild waterfowl are natural reservoirs for many IAVs. Almost all subtypes of avian IAVs (H1–H16 and N1–N9) have been identified in wild birds, and these birds have recently been implicated in the spread of HPAI H5N1 and H5N8 viruses. Waterfowl are an important host as they are usually asymptomatic and free-range ducks in wetland areas have considerable first-line contact with wild migratory birds, which aids the dissemination of influenza viruses to other birds. In this study, we characterized the genetic and antigenic properties of H3, H7, and H15 viruses isolated from free-range ducks in the Tanguar haor wetland area of Bangladesh; determined the pathogenicity of these viruses in a mouse model; and evaluated the pathogenicity and transmissibility of H7 viruses in a duck model.

We used DBA/2J mice to evaluate the disease potential of LPAI viruses from free-range ducks and wild birds in mammalian models. Several studies show that DBA/2J mice are more sensitive to influenza A infection than are other strains^27–29^. We previously used the DBA/2J mouse model to study the pathogenicity of North American H1N1 and H7 AIVs from migratory waterfowl^21, 28^. All AIVs used in the current study infect and replicate in mice without prior adaptation. However, H7N1 and H7N9 viruses isolated from free-range ducks caused higher mortality and increased pathogenicity than did H7N5 virus isolated from wild birds and other LPAI subtypes isolated from free-range ducks. In our previous studies, H7 AIVs of duck origin were more pathogenic than those of shorebird origin from the North American lineage^21^. Our current study confirmed this observation for viruses isolated from Eurasian lineages. Further studies focusing on replication kinetics in various avian and mammalian models are required to understand the mechanisms underlying the differences in replication characteristics.

Pathogenicity and transmissibility of HPAI and LPAI viruses via direct contact in different species of ducks have been previously reported^30–32^. The mallard is the duck model routinely used in our laboratory and is a good model for wild ducks^30, 33, 34^. We found that 2 avian H7 viruses infected donor ducks and ducks in direct contact with those donors, with no overt clinical signs of disease. Both the donor and direct contact ducks shed virus at higher titers from the cloaca than the trachea, which was expected given that most AIVs preferentially replicate in the gastrointestinal tract of wild ducks and are transmitted primarily via the fecal-oral route^32^.

H7 AIVs cause sporadic outbreaks in poultry populations worldwide. Epidemiological data on the H7N9 virus circulating in China indicate that it probably transferred from domestic ducks to chickens^10^. Recent studies also show that precursors of H7N3 and H7N8 HPAI viruses in the Americas most likely originated from dabbling ducks and diving ducks, respectively^35, 36^. It is considered that ducks can perpetuate most AIVs since they generally do not show clinical signs after infection. In this study, we provide evidence that free-range ducks can act as key intermediate hosts by acquiring and maintaining diverse influenza viruses from migratory birds. The H7 viruses evaluated in this study could not only replicate and cause disease in mammals but also be efficiently transmitted to mallard ducks in direct contact groups.

Phylogenetic analysis of *HA* genes of viruses isolated from the Tanguar haor wetland area of Bangladesh showed that they were of Eurasian lineage^19^. Molecular analysis of viruses revealed no change in four key highly conserved residues E190D, G225D, Q226L, and G228S (H3 numbering) in the receptor-binding site of the HA protein that have been associated with conferring avian-type specificity. Furthermore, these HAs did not contain a cleavage site associated with high pathogenicity, indicating that these viruses are classified as low pathogenic to chickens.

H15 influenza virus is rarely detected in wild birds, and was previously isolated from Australia from 1979 to 1983^37^ and more recently in Siberia and Ukraine^38, 39^. The H15 viruses are members of the subgroup that also includes H7N9 and H10N8 subtypes, which infected humans in 2013. We detected two H15 isolates from free-range ducks in our surveillance in Tanguar haor. Both viruses were genetically different from Australian viruses, but similar to those isolated from Ukraine and Siberia. We also found a specific insertion in the HA1 subunit at positions 253–262, which is characteristic of the *HA* gene of the H15 subtype^37^.

Our recent study showed the emergence of a new genotype of HPAI H5N1 viruses circulating in live poultry markets in Bangladesh. Multiple clades from HPAI H5N1 were detected in Bangladesh after the first H5N1 HPAI outbreak in 2007^16^. Routine surveillance showed that the H5N1 clade 2.3.2.1a viruses circulating in live poultry markets and poultry farms in Bangladesh have become the most dominant since 2011. The new genotype of HPAI H5N1 viruses contained three segments (HA, NA, and M) from clade 2.3.2.1a, and other segments were more closely related to those from LPAI viruses we isolated from Tanguar haor^19^, indicating that viruses isolated from Tanguar haor play an important role in the diversity of HPAI H5N1.

Future studies need to determine the effect of new segments that produce new genotypes on the pathogenicity and transmissibility of H5N1. The dynamics and transmission of AIVs through migratory wild birds are complex. Migratory birds infected with avian IAVs are (1) asymptomatic or display subclinical symptoms, (2) maintain the diversity of influenza viruses through various subtype combinations, and (3) allow the virus to spread over long distances. Therefore, monitoring AIVs in migratory wild birds is important for understanding the evolution and emergence of viruses with new biological properties, and surveillance of free-range ducks can be used as an early warning system for AIV outbreaks in poultry and to better understand the potential risk these viruses pose to humans. Our studies highlight the important role of free-range ducks in the spread of influenza viruses from migratory birds to live poultry markets, which is a potential risk to human and veterinary public health.

## Materials and methods

### Ethics statement

Experiments in mice were conducted in an Animal Biosafety Level 2+ facility at St. Jude Children’s Research Hospital (St. Jude), in compliance with the policies of the National Institutes of Health and Animal Welfare Act and with the approval of the St. Jude Institutional Animal Care and Use Committee. Ducks experiments were approved and performed according to guidelines set by the St. Jude Animal Care and Use Committee in an enhanced Animal Biosafety Level 3+ containment facility.

### Viruses

Thirteen AIVs were isolated from wild birds and free-range flocks of domestic ducks in the Tanguar haor region through collaborative efforts between the Center of Excellence for Influenza Research and Surveillance at St. Jude (Memphis, TN, USA) and Jahangirnagar University (Dhaka, Bangladesh) in February 2015 and February 2016. Four influenza A (H3N6), 4 influenza A (H7N1), one influenza A (H7N5), three influenza A (H7N9), and two influenza A (H15N9) viruses were isolated from free-range flocks of domestic ducks. Also, an influenza A (H7N5) virus was isolated from a migratory black-tailed godwit. All viruses were propagated and titrated for infectious dose in the allantoic cavities of 10-day-old embryonated chicken eggs at 35 °C for 48 h. Viral titers were determined by injecting 100 μL of serial 10-fold dilutions of the virus into allantoic cavities of 10-day-old embryonated chicken eggs and calculating the 50% EID_50_ according to the method of Reed and Muench^40^.

### Antigenic characterization

The hemagglutination inhibition (HI) assay was used to antigenically characterize the viruses. The H3N6, H7N1, H7N5, and H7N9 viruses were tested with polyclonal ferret and goat antiserum raised against representative H3, H7, and H15 viruses. Reference antisera against H3 and H7 viruses were selected from North American and Eurasian lineages. The four antisera against A/duck/Bangladesh/26920/2015 (H3N6), A/duck/Bangladesh/26980/2015 (H7N9), A/duck/Bangladesh/24692/2015 (H7N1), and A/duck/Bangladesh/24697/2015 (H15N9) were generated in the current study. Briefly, ferrets were first infected intranasally with 1 mL of 10^6^ EID_50_/mL of viruses and then boosted after 3 weeks by an intramuscular injection of virus with adjuvant. Blood was collected 1 week later for serum isolation. The HI test was performed according to WHO protocols^41^.

### Pathogenicity in DBA/2J mice

Seven isolates were selected to investigate pathogenicity in DBA/2J mice. Groups of 8 mice were anesthetized with isoflurane and inoculated intranasally with 30 µL of 10^6^ EID_50_/mL of virus. The uninfected (control) group was anesthetized and intranasally inoculated with 30 µL PBS pH 7.2. Upon virus challenge, five mice were monitored for 14 dpi for disease symptoms, weight loss, and mortality. Mortality was recorded on the basis of actual death or a 25% weight loss cutoff, according to our animal protocol. Three mice from each group were killed by CO_2_ administration and cervical dislocation after 3 days, and lung tissue was collected to determine viral titers. The total pathogenicity score was calculated as the sum of survival and weight loss scores (survival score 0.8 (survival area under the curve (AUC)/maximum AUC); weight loss score 0.2 (weight loss AUC/maximum AUC)), as described previously^28^. The mean weight loss of groups of five mice, each group inoculated with a different virus, was used for these calculations. As mortality was more important than morbidity in our screening, we used 80% weight for survival and 20% weight for weight loss after normalizing AUCs to the maximum AUC that could be obtained with no mortality or morbidity (uninfected group). The AUCs for survival and weight loss were calculated using GraphPad Prism. According to total pathogenicity scores, viruses were divided into five categories and assigned a PI value of 0 through 4, with 0 being nonpathogenic and 4 being the most pathogenic.

### Viral titers in lung and extrapulmonary tissues in DBA/2J mice

Groups of 12 mice were anesthetized with isoflurane and inoculated intranasally with 30 µL of 10^6^ EID_50_/mL of A/duck/Bangladesh/24692/2015 (H7N1) (PI-4), A/duck/Bangladesh/26980/2015 (H7N9) (PI-2), and A/black-tailed godwit/Bangladesh/24734/2015 (H7N5) (PI-0). Three mice per group were killed with CO_2_ at 2, 4, 6, and 8 dpi. Lung, intestine, liver, brain, and heart were collected to determine viral titers. All organs were homogenized in sterile PBS using the TissueLyser II (Qiagen, Gaithersburg, MD). Organ homogenates were centrifuged at 2000×*g* for 10 min, and supernatants were transferred to clean tubes. Viral titers were determined by the EID_50_ assay.

### Histology and immunohistochemistry

Lungs were collected at 2, 4, 6, 8 dpi, and fixed by intratracheal infusion and immersion, using a 10% neutral buffered formalin solution. Tissues were embedded in formalin, sectioned, and stained with hematoxylin and eosin, and immunohistochemistry (IHC) staining of serial histologic sections was performed to determine the distribution of influenza virus antigen. For IHC analysis, a goat primary polyclonal antibody (US Biological, Swampscott, MA) against anti-influenza A, USSR (H1N1) was used 1:1000 on tissue sections previously subjected to antigen retrieval for 30 min at 98 °C (Target Retrieval Solution [pH 9]; Dako Corp., Carpinteria, CA). To quantify the extent of viral infection in the lungs, digital images of whole lung sections stained for viral antigen were first captured using the Aperio ScanScope XT Slide Scanner (Leica Biosystems, Buffalo Grove, IL), and then both uninfected and virus-positive regions were manually outlined and areas were determined using ImageScope software (Leica Biosystems).

### Pathogenicity in chicken

The IVPI was determined in groups of SPF white leghorn chickens as described in the *WHO Manual on Animal Influenza Diagnosis and Surveillance*^41^. The IVPI index was the mean score per chicken per observation over a 10-day period to determine the virulence of influenza virus isolates in chickens.

### Pathogenicity and transmission in mallard ducks

Three donor ducks were infected with 0.5 mL of 10^6^ EID_50_/mL dilution of virus stock of A/duck/Bangladesh/24692/2015 (H7N1) and A/duck/Bangladesh/26980/2015 (H7N9) in PBS via the natural route. Two ducks inoculated with 0.5 mL sterile PBS were used as negative controls. To examine virus transmission, three naive ducks were added to each group at 3 dpi and they shared food and drinking water for 24 h (contact ducks) before being moved to a separate cage. Body weights were determined at days 3, 5, 7, 10, and 14 for donor and contact ducks. Ducks were observed for clinical signs over 14 days. Oropharyngeal and cloacal swabs were collected from all birds at days 3, 5, 7, 10, and 14 to determine virus shedding. Viral titers from oropharyngeal and cloacal swabs were determined by the EID_50_ assay. Blood samples were taken from donor and contact ducks on day 14 for serology analysis.

### Statistical analyses

All statistical analyses were performed by one-way analysis of variance in combination with Bonferroni’s multiple comparisons test by using GraphPad Prism 5.0 (GraphPad Software).

## Electronic supplementary material

Supplemental figure s1

Supplementary figure legends

Supplementary tables
