# Supplementary figures and images for: Replication and pathogenic potential of influenza A virus subtypes H3, H7, and H15 from free-range ducks in Bangladesh in mammals
Source: Emerg Microbes Infect. 2018 Apr 25;7:70. doi: 10.1038/s41426-018-0072-7 (PMC5915612; doi:10.1038/s41426-018-0072-7)

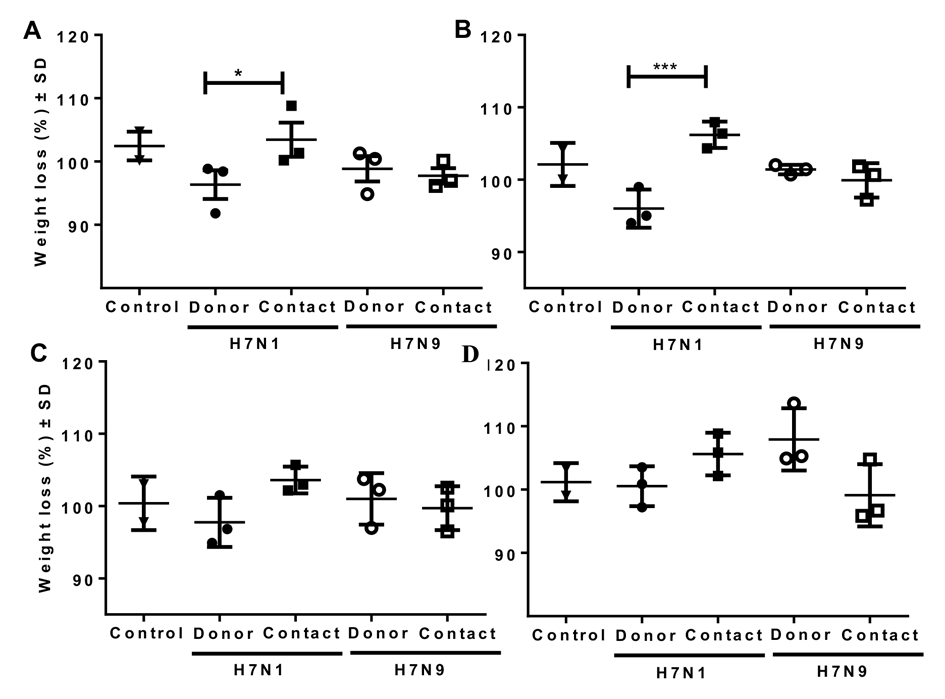

Supplement: Supplementary file 1 — Supplemental figure s1 [file 41426_2018_72_MOESM1_ESM.tif]
