# Supplementary material for: Replication and pathogenic potential of influenza A virus subtypes H3, H7, and H15 from free-range ducks in Bangladesh in mammals
Source: Emerg Microbes Infect. 2018 Apr 25;7:70. doi: 10.1038/s41426-018-0072-7 (PMC5915612; doi:10.1038/s41426-018-0072-7)
Supplement: Supplementary file 2 — Supplementary figure legends [file 41426_2018_72_MOESM2_ESM.docx]

**Supplemental Figure Legend**

**Figure S1** Percent weight loss in mallard ducks infected with H7N1 and H7N9 at 3, 5, 7, and 14 dpi. Bars represent standard deviations of the mean.
