# Supplementary material for: Replication and pathogenic potential of influenza A virus subtypes H3, H7, and H15 from free-range ducks in Bangladesh in mammals
Source: Emerg Microbes Infect. 2018 Apr 25;7:70. doi: 10.1038/s41426-018-0072-7 (PMC5915612; doi:10.1038/s41426-018-0072-7)
Supplement: Supplementary file 3 — Supplementary tables [file 41426_2018_72_MOESM3_ESM.docx]

**Supplemental table**

**Table S1 influenza viruses isolated from the Tanguar haor region of Bangladesh and IVPI.**

| **Strain** | **Subtype** | Host (Species) | **IVPI** |
| --- | --- | --- | --- |
| A/duck/Bangladesh/26920/2015 | H3N6 | domestic duck (*Anas sp*.) | 0.0 |
| A/duck/Bangladesh/26948/2015 | H3N6 | domestic duck (*Anas sp*.) | 0.05 |
| A/duck/Bangladesh/26974/2015 | H3N6 | domestic duck (*Anas sp*.) | ND |
| A/duck/Bangladesh/26918/2015 | H3N6 | domestic duck (*Anas sp*.) | 0.0 |
| A/duck/Bangladesh/24694/2015 | H7N1 | domestic duck (*Anas sp*.) | 0.31 |
| A/duck/Bangladesh/24692/2015 | H7N1 | domestic duck (*Anas sp*.) | 0.0 |
| A/duck/Bangladesh/24706/2015 | H7N1 | domestic duck (*Anas sp*.) | ND |
| A/duck/Bangladesh/24705/2015 | H7N1 | domestic duck (*Anas sp*.) | 1.04 |
| A/black-tailed godwit/Bangladesh/24734/2015 | H7N5 | black-tailed godwit (*Limosa limosa*) | 0.0 |
| A/duck/Bangladesh/26980/2015 | H7N9 | domestic duck (*Anas sp*.) | 0.0 |
| A/duck/Bangladesh/26992/2015 | H7N9 | domestic duck (*Anas sp*.) | 0.35 |
| A/duck/Bangladesh/27042/2015 | H7N9 | domestic duck (*Anas sp*.) | 0.0 |
| A/duck/Bangladesh/24704/2015 | H15N9 | domestic duck (*Anas sp*.) | ND |
| A/duck/Bangladesh/24697/2015 | H15N9 | domestic duck (*Anas sp*.) | 0.0 |

IVPI, intravenous pathogenicity index; ND, not done.

**Table S2 Antigenic characterization by the hemagglutination inhibition assay of H7 viruses isolated from free-range ducks in Bangladesh**

|  | **Monoclonal antibodies** | | | | | |
| --- | --- | --- | --- | --- | --- | --- |
|  | **A/chicken/Victoria/85 H7N7** | | **A/**S**eal/MA/1/80 H7N7** | | | |
|  |  |  | **I** | | **II** | |
| Reference virus | 4/2 | 14/1 | 55/2 | 58/2 | 46/6 | 71/6 |
| A/Seal/MA/1/80 H7N7 | 6400 | 400 | 6400 | 1600 | 6400 | 3200 |
| RG-A/Anhui/1/2013 H7N9 | 200 | 400 | 12800 | 3200 | 6400 | 3200 |
| RGA/Netherlands/219/2003H7N7 | 3200 | 400 | 6400 | 1600 | 3200 | 800 |
| RG-A/Canada/RV444/2004 H7N3 | 3200 | 400 | 6400 | 800 | 6400 | 400 |
| Test virus |  |  |  |  |  |  |
| A/duck/Bangladesh/24692/2015 H7N1 | 3200 | 400 | 3200 | 800 | <100 | 100 |
| A/duck/Bangladesh/26980/2015 H7N9 | 3200 | 400 | 6400 | 400 | 3200 | 100 |
| A/duck/Bangladesh/26992/2015 H7N9 | 3200 | 400 | 3200 | 200 | <100 | 200 |
| A/duck/Bangladesh/27042/2015 H7N9 | 6400 | 400 | 12800 | 400 | 6400 | 200 |
| A/duck/Bangladesh/24694/2015 H7N1 | 6400 | 400 | 6400 | 1600 | 6400 | 200 |
| A/duck/Bangladesh/24705/2015 H7N1 | 6400 | 800 | 12800 | 1600 | 6400 | 200 |
| A/duck/Bangladesh/24706/2015 H7N1 | 6400 | 400 | 6400 | 1600 | <100 | 200 |
| A/black-tailed godwit/Bangladesh/24734/2015 H7N5 | 6400 | 400 | 6400 | 1600 | 3200 | 100 |

**Table S3 Antigenic characterization of H15 viruses isolated from free-range ducks in Bangladesh**

|  | **Antisera** | |
| --- | --- | --- |
|  | **Australia** | **Bangladesh** |
| Reference virus | A/duck/Australia/341/1983(H15N8) | A/duck/Bangladesh/24697/2015(H15N9) |
| A/Australian shelduck/Western Australia/1756/1983(H15N2) | 20 | 20 |
| A/shearWater/Australia/2576/1979(H15N9) | 20 | 20 |
| A/duck/Australia/341/1983(H15N8) | **320** | 20 |
| A/sooty tern/Western Australia/2190/1983(H15N9) | 40 | 20 |
| A/Australian shelduck/Western Australia/1762/1979(H15N9) | 40 | 20 |
| A/wedge-tailed shearwater/Western Australia/2327/1983(H15N9) | 20 | 20 |
| Test virus |  |  |
| A/duck/Bangladesh/24697/2015(H15N9) | 20 | **80** |
| A/duck/Bangladesh/24704/2015(H15N9) | ND | 80 |

ND, not done.

**Table S4** **Clinical signs, mortality, and seroconversion (hemaggultinin inhibition titers) in mallard ducks**

| **Virus** | **Group** | **Clinical signs** | **Mortality** | **Seroconversion (log_2_, mean ±SD)** |
| --- | --- | --- | --- | --- |
| A/duck/Bangladesh/24692/2015 (H7N1) | Donor | 0/3 | 0/3 | 3/3 (5.320±1.0) |
|  | Contact | 0/3 | 0/3 | 3/3 (3.65±0.57) |
| A/duck/Bangladesh/246980/2015 (H7N9) | Donor | 0/3 | 0/3 | 3/3 (4.32±1.0) |
|  | Contact | 0/3 | 0/3 | 3/3 (3.32±0.0) |

SD, standard deviation.
